# The role of ion homeostasis in adaptation and tolerance to acetic acid stress in yeasts

**DOI:** 10.1093/femsyr/foae016

**Published:** 2024-04-24

**Authors:** Miguel Antunes, Isabel Sá-Correia

**Affiliations:** iBB—Institute for Bioengineering and Biosciences, Instituto Superior Técnico, Universidade de Lisboa, 1049-001, Lisbon, Portugal; Department of Bioengineering, Instituto Superior Técnico, Universidade de Lisboa, 1049-001, Lisbon, Portugal; Associate Laboratory i4HB—Institute for Health and Bioeconomy at Instituto Superior Técnico, Universidade de Lisboa, 1049-001, Lisbon, Portugal; iBB—Institute for Bioengineering and Biosciences, Instituto Superior Técnico, Universidade de Lisboa, 1049-001, Lisbon, Portugal; Department of Bioengineering, Instituto Superior Técnico, Universidade de Lisboa, 1049-001, Lisbon, Portugal; Associate Laboratory i4HB—Institute for Health and Bioeconomy at Instituto Superior Técnico, Universidade de Lisboa, 1049-001, Lisbon, Portugal

**Keywords:** ion fluxes, H^+^ homeostasis, K^+^ homeostasis, stress tolerance, response to stress, weak acids

## Abstract

Maintenance of asymmetric ion concentrations across cellular membranes is crucial for proper yeast cellular function. Disruptions of these ionic gradients can significantly impact membrane electrochemical potential and the balance of other ions, particularly under stressful conditions such as exposure to acetic acid. This weak acid, ubiquitous to both yeast metabolism and industrial processes, is a major inhibitor of yeast cell growth in industrial settings and a key determinant of host colonization by pathogenic yeast. Acetic acid toxicity depends on medium composition, especially on the pH (H^+^ concentration), but also on other ions’ concentrations. Regulation of ion fluxes is essential for effective yeast response and adaptation to acetic acid stress. However, the intricate interplay among ion balancing systems and stress response mechanisms still presents significant knowledge gaps. This review offers a comprehensive overview of the mechanisms governing ion homeostasis, including H^+^, K^+^, Zn^2+^, Fe^2+/3+^, and acetate, in the context of acetic acid toxicity, adaptation, and tolerance. While focus is given on *Saccharomyces cerevisiae* due to its extensive physiological characterization, insights are also provided for biotechnologically and clinically relevant yeast species whenever available.

## Introduction

Yeast cells must have the ability to quickly and effectively respond to sudden environmental challenges to ensure their fitness and survival, a trait extensively explored in the yeast model and cell factory *Saccharomyces cerevisiae* (Estruch 2000, López-Maury et al. 2008, Saini et al. 2018). Central to yeast’s stress response is the maintenance of ionic homeostasis, where dissimilarities in ion concentrations across cellular membranes establish crucial gradients for proper cellular function (Orij et al. 2011, Ke et al. 2013, Yenush 2016, Antunes et al. 2023). These ionic gradients, which define the transmembrane electrochemical potential, are fundamental for maintaining cellular physiology, solute translocation, and energy homeostasis, preventing toxicity from intracellular surplus ion concentrations (Canadell and Ariño 2016). Perturbations to any of these gradients, especially under stress, can substantially disrupt the overall electrochemical transmembrane potential and the balance of other ions, underscoring the interconnectedness of ion homeostasis, cellular function, and stress tolerance (Orij et al. 2011). Despite significant progress in understanding yeast ion fluxes and homeostasis, knowledge gaps remain regarding regulatory mechanisms, their intracellular distribution, and the interplay of different ions within the cell, especially under stress conditions.

Acetic acid constitutes a prime example of an environmental stress factor that directly challenges ion homeostasis (Macpherson et al. 2005, Mira et al. 2010b, Wan et al. 2015, Palma et al. 2018, Xu et al. 2020, Guaragnella and Bettiga 2021). This short-chain weak organic acid, despite being a carbon source for several yeast species, is a toxic byproduct of alcoholic fermentation and a widely used food preservative (Mira et al. 2010b, Palma et al. 2018, Cunha et al. 2019). The impact of its effects extends to its prevalence as a major inhibitor in various industrial settings, and to the pathogenicity of yeast species (Cunha et al. 2017, 2019, Ullah et al. 2013b, Deparis et al. 2017). In industrial settings, acetic acid drives the need for the pursuit and engineering of robust stress-tolerant yeast species to enhance bioproduct and biomass yields, particularly as demand grows for sustainable and green chemicals (Deparis et al. 2017). Moreover, understanding yeast responses to acetic acid is crucial in the context of invasive fungal infections, where the ability to withstand this stress can influence pathogenic *Candida* species ability to thrive and survive during host colonization (Moosa et al. 2004, Ullah et al. 2013b, Cunha et al. 2017, Lourenco et al. 2019).

The regulation of intracellular pH (pHi) is a paradigmatic example of the critical nature of ion homeostasis in yeast response to acetic acid-induced toxicity, highly dependent on the medium pH (i.e. H^+^ concentration) (Orij et al. 2011, Stratford et al. 2013b, Ullah et al. 2013b, Antunes et al. 2023). Low pH values in the medium enhance the effectiveness of microbial growth inhibition by acetic acid (Pampulha and Loureiro-Dias 1989, Mira et al. 2010b, Palma et al. 2018). This effect results from the balance between the protonated and dissociated forms of acetic acid: as the medium pH decreases from the acetic acid pKa value of 4.75, the concentration of undissociated acetic acid (CH_3_COOH) toxic form increases (Arneborg et al. 2000). Notably, acetic acid possesses an interesting feature: it can act as both a hydrogen bond donor and acceptor, enabling the formation of stable dimeric complexes in nonpolar solvents (Pem et al. 2023). This characteristic presumably allows for passive diffusion of the undissociated acid in the form of dimeric complexes across the nonpolar interior of yeast plasma membrane lipids. Acetic acid uptake was also proposed to be facilitated by the aquaglyceroporin Fps1 (Mollapour and Piper 2007). Raising the medium pH above the pKa value of acetic acid leads to a higher abundance of acetate anions. In Crabtree-positive yeast species, like *S. cerevisiae*, when acetic acid is the sole carbon source or when glucose or another repressing carbon source is absent, these charged counterions can enter the cell via a H^+^-symport mechanism mediated by secondary active monocarboxylic acid transporters such as Ady2 and Jen1 (Casal et al. 1996, 2016).

Upon encountering a near-neutral pH in the cytosol, acetic acid dissociates into the acetate anion (CH_3_COO^−^), accompanied by proton release (Arneborg et al. 2000, Mira et al. 2010b, Palma et al. 2018). Since the dissociated acid form cannot escape to the extracellular medium through passive diffusion due to its charge, this leads to its intracellular accumulation, interfering with cellular metabolism and causing oxidative stress (Almeida et al. 2009, Semchyshyn et al. 2011). On the other hand, increased intracellular proton concentration leads to cytosol acidification and dissipation of the transmembrane proton gradient (Pampulha and Loureiro-Dias 1990, Ding et al. 2013). To mitigate the effects of acetic acid stress, yeast cells trigger the ATP-dependent efflux of protons and acetate from the cell (Carmelo et al. 1997, Sá-Correia and Godinho 2022, Zhang et al. 2022). However, this active efflux results in a significant depletion of the cellular ATP pools, reflected in the reduction in yeast maximum biomass yield and specific growth rate observed under acetic acid stress (Pampulha and Loureiro-Dias 2000). Moreover, cellular envelope rearrangements, including alterations in cell wall structure and plasma membrane lipid composition, are crucial to regulate the stability and function of relevant transport proteins and, to reduce the rate of diffusion of the undissociated acid form back into the cell. This effectively counteracts the futile cycle created by the active efflux of acetate and protons if followed by the re-entry of the liposoluble acid form (Van Der Rest et al. 1995, Ullah et al. 2013a, Ribeiro et al. 2021). Additionally, the presence and nature of nutrients (carbon and nitrogen sources, amino acids, and vitamins) and minerals (such as K^+^, Fe^2+/3+^, and Zn^2+^) also contributes to yeast adaptation to acetic acid-induced stress (Macpherson et al. 2005, Mira et al. 2010b, Wan et al. 2015, Zhang et al. 2017, Xu et al. 2019b, Castillo-Plata et al. 2022, Chen et al. 2022, Antunes et al. 2023).

**Figure 1.**
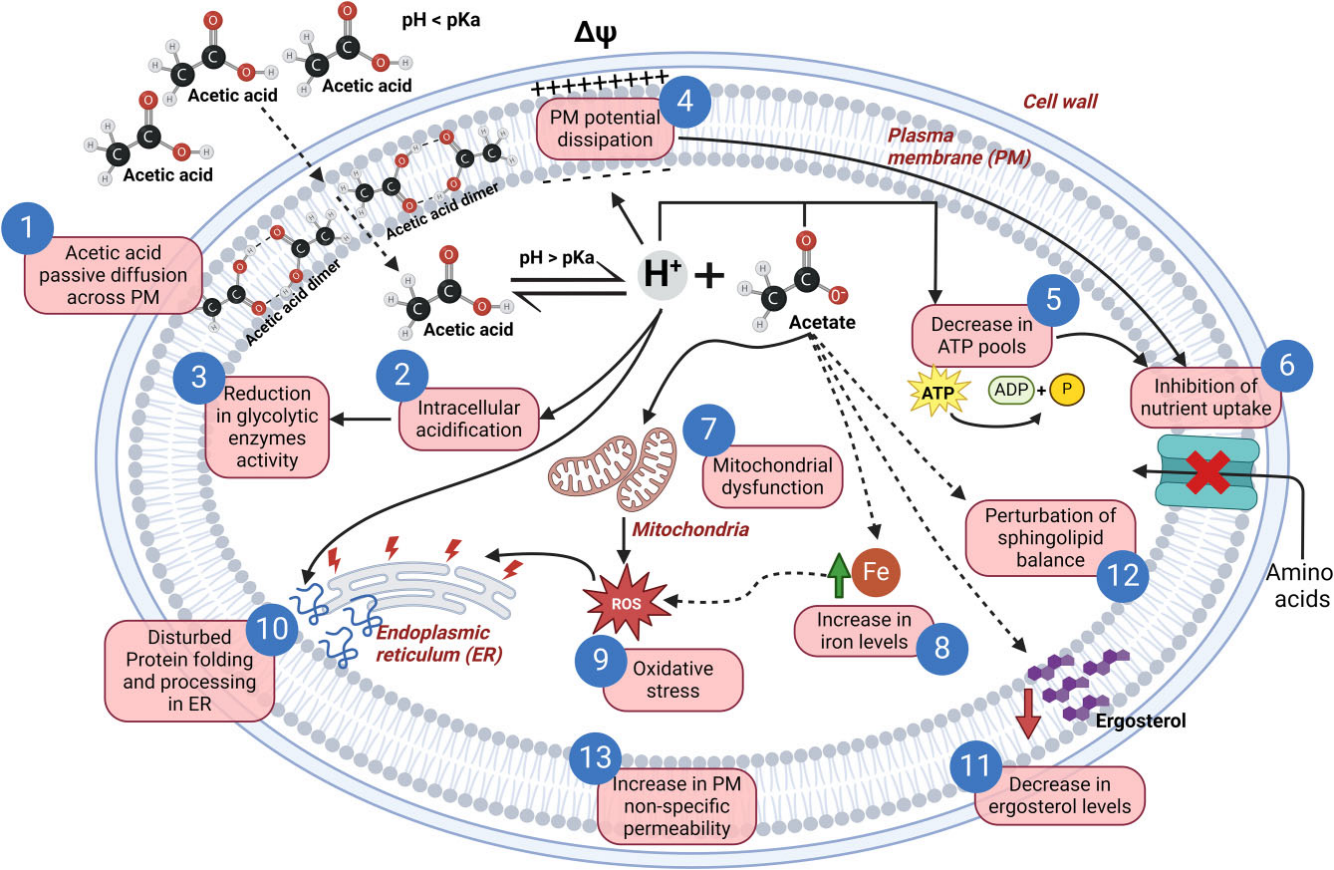
Illustration of the mechanisms of toxicity induced by acetic acid in *S. cerevisiae*. Brief descriptions of each of the enumerated acetic acid toxicity processes are provided in Table 1.

**Figure 2.**
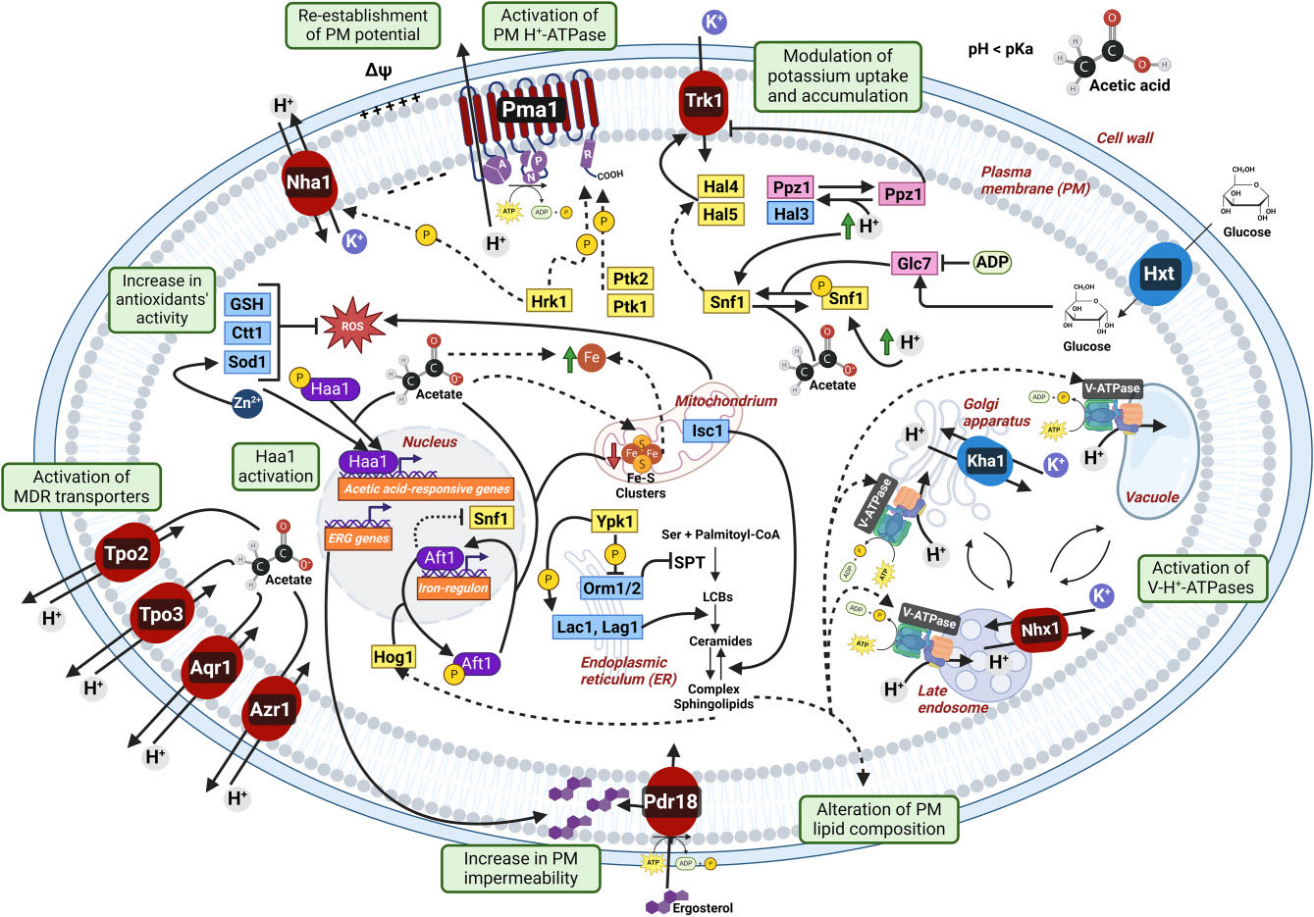
Illustration of the mechanisms of response and adaptation, reported and proposed for *S. cerevisiae*, to acetic acid stress through the regulation of ion homeostasis and fluxes. Some of these mechanisms are included in Table 1. Kinases and phosphatases are displayed in yellow and pink, respectively. Transcription factors are displayed in purple. Membrane proteins reported to be associated with acetic acid stress tolerance are represented in red. Pma1 is shown in the monomeric form to convey the mechanisms of regulation. Dashed arrows refer to putative or indirect associations.

This review explores the physiological mechanisms behind ion homeostasis in the context of acetic acid toxicity, adaptation, and tolerance. It provides an overview of the roles and regulation of the balance and fluxes across cellular membranes of key ions involved in the corresponding stress response, such as protons (H^+^), potassium (K^+^), iron (Fe^2+/3+^), zinc (Zn^2+^), and acetate (CH_3_COO^−^). By examining the genes and proteins involved in stress-signaling pathways that mediate these ions’ regulation, this review aims to elucidate the complex strategies employed by yeasts to maintain ion balance and ensure efficient adaptation and survival under acetic acid stress. This information is extensive for *S. cerevisiae* since the molecular mechanisms underlying the response to this stress have been extensively characterized in this species (Palma et al. 2018, Guaragnella and Bettiga 2021). However, whenever available, this review also incorporates and provides insights on the topic in relevant biotechnological and clinical yeast species. Two accompanying figures are provided, displaying an overview of acetic acid toxicity mechanisms (Fig. 1) and adaptation strategies (Fig. 2) in *S. cerevisiae* from the context of intracellular ion balances and fluxes.

**Table 1.** Brief descriptions of the mechanisms linked to the processes underlying acetic acid toxicity, as enumerated in Fig. 1. These descriptions may also refer to mechanisms of response and adaptation to acetic acid, as depicted in Fig. 2, where necessary, for clarity.

Process	Mechanism	References
(**1**) Acetic acid passive diffusion	Acetic acid in its undissociated form (when extracellular pH *< *pKa) crosses the PM through passive diffusion, likely adopting a dimeric structure	Casal et al. (1996), Mollapour and Piper (2007), Lindahl et al. (2018), Pem et al. (2023)
(**2**) Intracellular acidification	Cytosolic dissociation of acetic acid (where pH *> *pKa) leads to the accumulation of protons and subsequent intracellular acidification	Pampulha and Loureiro-Dias (1989), Arneborg et al. (2000), Antunes et al. (2023)
(**3**) Reduction in glycolytic flux	Cytosol acidification inhibits the activity of several glycolytic enzymes	Pampulha and Loureiro-Dias (1990), Almeida et al. (2009), Stratford et al. (2013a), Dong et al. (2017)
(**4**) PM potential dissipation	The accumulation of protons intracellularly leads to the depolarization of plasma membrane through the dissipation of the H^+^ gradient	Godinho et al. (2018), Antunes et al. (2023)
(**5**) Decrease in ATP pools	Activation of the ATP-dependent efflux of protons, through the PMH^+^-ATPase Pma1, and of acetate, via MDR/MXR transporters (Fig. 2), results in a depletion of cellular ATP pools	Pampulha and Loureiro-Dias (2000), Fernandes et al. (2005), Ullah et al. (2013a), Zhang et al. (2022)
(**6**) Inhibition of nutrient uptake	A reduction in intracellular ATP pools, coupled with disruptions in proton gradients, hinders the ATP-dependent symport of nutrients such as amino acids	Almeida et al. (2009), Ding et al. (2013), Dong et al. (2017)
(**7**) Oxidative stress	Acetate has been shown to have a prooxidant effect, leading to activation of the antioxidative response (Fig. 2)	Semchyshyn et al. (2011), Guaragnella et al. (2019)
(**8**) Disturbed protein folding and processing in ER	Acetate induces endoplasmic reticulum (ER) stress and triggers the unfolded protein response (UPR). Additionally, intracellular acidification leads to protein denaturation	Dong et al. (2017), Kawazoe et al. (2017)
(**9**) Mitochondrial dysfunction	Acetic acid exposure causes acidification of the mitochondrial matrix and induces mitochondrial structural alterations and degradation	Rego et al. (2012), Dong et al. (2017)
(**10**) Perturbation of iron homeostasis	Acetic acid stress exposure leads to increased expression of genes involved in iron uptake, translocation of Aft1 to the nucleus (Fig. 2), and elevated intracellular iron levels	Kawahata et al. (2006), Mira et al. (2010b), Martins et al. (2018)
(**11**) Decrease in ergosterol levels	Exposure to acetic acid stress leads to a decrease in ergosterol levels and induces the expression of genes involved in ergosterol biosynthesis and *PDR18*, which encodes a PM ABC transporter essential for preserving optimal ergosterol levels in the PM (Fig. 2)	Lindberg et al. (2013), Godinho et al. (2018), Ribeiro et al. (2022)
(**12**) Perturbation of sphingolipid balance	Acetic acid stress induces alterations in sphingolipid content, affecting cell fate, protein trafficking, signaling pathways and membrane permeability	Rego et al. (2012, 2018)
(**13**) Increase in plasma membrane nonspecific permeability	Exposure to acetic acid destabilizes the PM, disrupting proper selective permeability. As a response mechanism, alterations of plasma membrane lipid composition restrict the passive diffusion of lipophilic toxic compounds into the cell (Fig. 2)	Godinho et al. (2018), Ribeiro et al. (2022)

## Homeostasis and regulation of proton (H^+^) fluxes and the response and tolerance to acetic acid stress

### The plasma membrane and organellar H^+^-ATPases as the main regulators of H^+^ fluxes and homeostasis

Plasma membrane proton gradient in yeast is generated by the activity of plasma membrane H^+^-ATPase Pma1 (Morsomme et al. 2000, Kane 2016, Zhao et al. 2021). This H^+^ gradient serves as a primary driving force supporting active transport of ions, nutrients, and metabolites through H^+^-coupled transporters, which use this gradient to move solutes against their concentration gradients (Kane 2016). *PMA1* is an essential gene; mutations that result in defects in Pma1 activity commonly lead to reduced specific growth rates and resistance to hygromycin B, an indicator of plasma membrane depolarization (Serrano et al. 1986, Perlin et al. 1988). The active H^+^ pumping activity of Pma1 accounts for a significant portion of cellular ATP consumption during growth on glucose and under acetic acid stress (Pampulha and Loureiro-Dias 2000, Ullah et al. 2013a). Pma1 is the main modulator of cytosolic pH (Serrano et al. 1986, Carmelo et al. 1997). Intracellular pH homeostasis is achieved through the continuous regulation of both cytosolic and organellar pH levels (Canadell and Ariño 2016). The pH within any given organelle is determined by a complex interplay involving passive proton leaks alongside the function of various pumps and channels (Brett et al. 2005, Kane 2016, Ariño et al. 2019). This regulation of organelle pH is crucial not only for maintaining the internal environment of the organelle but also plays a role in the broader cellular dynamics, including vesicle trafficking among organelles within the secretory and endosomal pathways (Brett et al. 2005, Kane 2016).

Another notable player in pHi homeostasis is the vacuolar-type proton-translocating ATPases (V-ATPases). They consist of multisubunit enzyme complexes divided into two main sectors: V_1_ and V_0_, each composed of several subunits (Kane 2007). The V_1_ sector, facing the cytoplasm, harbors the catalytic ATPase activity, while the V_0_ sector, embedded in the membrane of organelles (vacuole/lysosomes, endosomes, and Golgi) is responsible for proton translocation (Kane 2007). As ATP-driven proton pumps, their primary function involves transporting protons from the cytoplasm into these organelles (reviewed in Kane 2007). This proton transport is crucial for maintaining the acidic pH of diverse organelles, facilitating various cellular processes, including protein sorting, nutrient storage, and detoxification. Remarkably, unlike most eukaryotes, deletion of genes coding for any of the subunits or assembly factors (*vma* mutants) in *S. cerevisiae* does not lead to lethality under nonstressing conditions. However, it renders cells extremely sensitive to pH fluctuations, unable to grow on nonfermentable carbon sources, and susceptible to stress induced by a wide range of inhibitors, in particular acetic acid and other weak acids, and heavy metals (Kawahata et al. 2006, Kane 2007, Mira et al. 2010b, Tarsio et al. 2011, Deprez et al. 2021, Mota et al. 2024).

### Regulation of Pma1 under acetic acid stress

The essential protein Pma1 is composed of 10 transmembrane helices and three cytosolic domains: the actuator domain (A), the nucleotide binding domain (N), and the phosphorylation domain (P). Furthermore, the C-terminal tail of the H^+^-ATPase, known as the regulatory (R) domain, plays a crucial role in modulating Pma1 activity (Kane 2016, Zhao et al. 2021). This domain can interact with the P domain to autoinhibit the H^+^-pump activity (Kane 2016, Zhao et al. 2021). Pma1 is highly abundant in the plasma membrane, representing at least 15% of all plasma membrane proteins (Serrano 1988). It has been shown to oligomerize, forming hexamers, a process influenced by sphingolipids and to not be homogeneously distributed across the PM but instead accumulating in a membrane subcompartment denominated MCP (membrane compartment containing Pma1) (Zhao et al. 2021). This MCP is characterized by being depleted in ergosterol and exclusively enriched in sphingolipids (Zhao et al. 2021).

The activation of Pma1 is among the cellular responses when cells are exposed to acetic acid stress (Carmelo et al. 1997, Stratford et al. 2013a, Antunes et al. 2023). Under this stress, Pma1 actively pumps protons out of the cell, helping to restore pHi up to more physiological values and preventing further acidification and potential harm to the cell (Carmelo et al. 1997). Even though the activation of Pma1 at low pH is glucose-dependent due to the continuous requirement of cytosolic ATP, the mechanism of Pma1 activation in this condition apparently differs from the one observed for the well-described glucose-induced activation of Pma1 (Mazón et al. 2015). Pma1 activation by glucose metabolism is reversible and implicates a shift of the optimum pH to more alkaline values, whereas activation in cells exposed to the weak organic acid, succinic acid, results in an irreversible change, i.e. not associated with an alteration of the optimum pH but is dependent on the growth phase (Eraso and Gancedo 1987, Benito et al. 1992, Mazón et al. 2015). Both these activation mechanisms have been shown to result in an increase in affinity for ATP and to require the C-terminal domain of Pma1 (Eraso and Gancedo 1987, Benito et al. 1992, Mazón et al. 2015). At low pH values, stimulation of Pma1 is associated with a conformational change and potential destabilization of salt bridges between the R and P domains (Zhao et al. 2021). In *S. cerevisiae*, three phosphorylation residues within the R domain have been identified as relevant, contributing to the relieve of autoinhibitory interactions between the R and P domains: Ser899, whose phosphorylation correlates with a higher affinity of Pma1 for ATP; and Ser911 and Thr912, consisting of two adjacent residues whose phosphorylation leads to an increase in V*_max_* and is crucial for the full activation of Pma1 (Portillo 2000, Eraso et al. 2006, Mazón et al. 2015). The phosphorylation state of these residues is so critical that mutations of *PMA1* at these sites (S911A–T912A) can lead to a substantial reduction in Pma1 activity, impeding cell growth (Guarini et al. 2024). Specific yeast protein kinases play an instrumental role in the activation of Pma1 through phosphorylation. The protein kinase Hrk1 is a major acetic acid stress tolerance determinant in yeast (Antunes et al. 2018, 2023, Mira et al. 2010b, Bernardo et al. 2017, Guerreiro et al. 2017, Xu et al. 2019a), belonging to the NPR/Hal family (Antunes and Sá-Correia 2022). Under acetic acid stress conditions, Hrk1 contributes to the phosphorylation of Pma1 residues Ser911 and Thr912 (Guerreiro et al. 2017), whereas in response to glucose metabolism the kinases Ptk1 and Ptk2 act both in a largely redundant manner in mediating the phosphorylation of Ser911 and Thr912, with Ptk2 promoting the double phosphorylation more efficiently than Ptk1 (Guarini et al. 2024). Although Hrk1 contributes to enhancing Pma1 activity under acidic conditions (Antunes et al. 2023), where it is presumed to be most active, its role in activating this proton pump in response to glucose metabolism appears to be minimal or undetectable (Goossens et al. 2000, Antunes et al. 2023, Guarini et al. 2024). Further investigation is needed to elucidate the putative roles of kinases Ptk1 and Ptk2, as well as to identify potential involvement of other kinases and the mechanisms underlying Hrk1 activity in the activation of Pma1 under acetic acid stress. Interestingly, the activity of Pma1 stimulated by an increase in cytosolic H^+^ concentration can trigger a signaling cascade leading to TORC1 activation (Guarini et al. 2024). For instance, the transport of amino acids via H^+^ symporters, causing a local increase in cytosolic H^+^ concentration, can stimulate Pma1 activity (Guarini et al. 2024). This enhanced activity might then contribute to TORC1 activation through mechanisms that are yet to be fully understood. Conversely, the activation of TORC1 appears to feedback-inhibit Pma1 phosphorylation at the phosphorylation sites Ser911–Thr912 (Guarini et al. 2024). Notably, it has been proposed that Hrk1 might be involved in the stimulation of Ptk1 and Ptk2 in response to H^+^ increase, based on the observation that the Pma1 residues Ser911 and Thr912 exhibit less pronounced phosphorylation upon H^+^-coupled amino acid uptake in the *hrk1*∆ deletion mutant strain, and that Hrk1 is unable to sustain the phosphorylation of these residues in *ptk1*∆*ptk2*∆ cells (Guarini et al. 2024). Notably, the activity of Pma1 is reduced in *vma* mutants, resulting either from Pma1 internalization, as a compensatory mechanism, or due to limitations in its activity through yet unidentified mechanisms (Kane 2016, Wilms et al. 2017). V-ATPase activity is crucial for yeast adaptation to acetic acid stress, as *vma* mutants are not able to recover the initial pHi drop induced by this acid and resume growth (Carmelo et al. 1997, Kawahata et al. 2006, Mira et al. 2010b, Tarsio et al. 2011, Wilms et al. 2017, Deprez et al. 2021, Mota et al. 2024). Overall, the coordination of Pma1 and V-ATPase activity represents a vital mechanism for maintaining pH homeostasis within the cell, in the absence or presence of acetic acid stress.

### Initial pHi of individual cells influences yeast ability to adapt to sudden acetic acid stress

Cell-to-cell heterogeneity is a relevant parameter to consider in the context of a yeast culture adaptation to sudden acetic acid-induced stress. The adaptation to acetic acid stress is partially associated to the initial pHi of individual cells. Lower initial pHi values in a subpopulation of *S. cerevisiae* cells were reported to prevent sharp drops in pHi following acetic acid exposure since the resulting intracellular concentration of dissociated acid is presumably lower in these cells, enhancing the likelihood of resuming proliferation (Fernández-Niño et al. 2015). These mechanisms are still fairly uncharacterized, thus an explanation for this phenomenon may also be attributed to other intrinsic physiological factors, such as higher basal or acetic acid-induced H^+^-ATPase pumps activity, more efficient acetate efflux systems, or higher plasma membrane impermeability in those cells (Fernández-Niño et al. 2015).

The importance of the relationship between pH homeostasis and acetic acid tolerance is evidenced by the differences in short-term alterations of pHi upon acetic acid stress exposure in other yeast species. Measurements of pHi in *C. glabrata* exposed to acetic acid stress revealed that this acid causes a similar decrease in pHi as the one reported in *S. cerevisiae* cells and that the recovery of pHi in both species is similar, following adaptation to acetic acid stress, although *S. cerevisiae* appears to be more tolerant to this stress condition (Ullah et al. 2013b). Differences among *C. glabrata* strains in tolerance to acetic acid stress have been attributed to a higher activity of the plasma membrane H^+^-ATPase CgPma1 and a diminished accumulation of intracellular acetate (Cunha et al. 2017). The highly acetic acid tolerant yeast species, *Zygosaccharomyces bailii*, exhibits a remarkable tolerance to short-term decreases in pHi (Arneborg et al. 2000, Dang et al. 2012). This difference has been proposed to be a result of a high impermeability of *Z. bailii* plasma membrane to the undissociated acetic acid form, a high buffering capacity of *Z. bailii* cytosol, and/or a higher amount of energy reserves available for the stress response compared to *S. cerevisiae* (Arneborg et al. 2000). Furthermore, *Z. bailii* was also shown to be able to temporarily tolerate a large drop in pHi induced by high inhibitory acetic acid concentrations during the exponential phase, which is only restored once cell proliferation ceases (stationary phase) (Dang et al. 2012). The high acetic acid tolerance phenotype of *Z. bailii* has been proposed to be in part due to the existence of a subpopulation of tolerant cells that have a basal lower pHi (from 0.4 to 0.8 pH units) than the acid-sensitive bulk population, resulting in a lower proportion of intracellular dissociation of acetic acid (Stratford et al. 2013b).

### The putative role of Snf1 kinase in acetic acid stress adaptation as an energy status- and pH-sensor

Cells lacking the *SNF1* gene, encoding the Snf1 kinase, demonstrate significant sensitivity to acetic acid stress (Mira et al. 2010b). This kinase plays a crucial role in modulating the expression of genes responsible for the catabolism of alternative carbon sources in yeast, particularly under conditions of glucose starvation (Simpson-Lavy and Kupiec 2022, 2023). Recent studies have expanded our understanding on Snf1’s function in this yeast species, revealing that, in addition to being an energy sensor, it also has a pH-sensing module, identified as a poly-histidine (polyHIS) tract located in the prekinase region (Simpson-Lavy and Kupiec 2022). Full activation of Snf1 in response to glucose deprivation is achieved through three independent mechanisms: phosphorylation at the T210 residue, protonation of the polyHIS tract, and de-SUMOylation of the K549 residue (Simpson-Lavy and Kupiec 2022). Changes in pHi levels directly influence the protonation state of the polyHIS tract; when pHi values become more alkaline, the polyHIS tract undergoes deprotonation, whereas acidic pHi values lead to its protonation, contributing to its activation (Simpson-Lavy and Kupiec 2022). The phosphorylation of Snf1 at T210 occurs, intriguingly, even in the presence of glucose, when yeast cells are exposed to acetic acid stress (Mira et al. 2010b), a phenomenon that could reflect Snf1’s role in sensing the cell’s energy status. This hypothesis can be rationalized by considering that ADP is the key metabolite activating Snf1 under conditions of glucose depletion (Mayer et al. 2011). Under acetic acid stress, there is an increase in the activity of plasma membrane H^+^-ATPase (Pma1) (Carmelo et al. 1997, Antunes et al. 2023), leading to heightened ATP consumption and potentially elevated ADP levels (Pampulha and Loureiro-Dias 2000). This suggests that acetic acid-induced ATP depletion and the presumable subsequent increase in ADP levels could serve as the primary driver behind Snf1 phosphorylation under these conditions. In addition to T210 phosphorylation, the drop in cytosolic pH caused by acetic acid stress is expected to lead to the protonation of the polyHIS tract, thereby increasing Snf1 activity. Overall, these observations suggest a complex interplay between Snf1 activity and acetic acid stress, pointing to a sophisticated regulatory network where Snf1 integrates signals from both the cellular energy state (phosphorylation of T210) and the pHi (polyHIS protonation). Further research is necessary to fully elucidate these relationships and the precise mechanisms through which Snf1 coordinates cellular responses to acetic acid stress. The role of Snf1 in the response and adaptation to acetic acid stress is probably not only limited to intracellular H^+^ concentration sensing, but also comprises the modulation of potassium and iron homeostasis, as outlined in the corresponding sections.

## Homeostasis and regulation of potassium (K^+^) fluxes and the response and tolerance to acetic acid stress

While Pma1 greatly contributes to the regulation of pHi and plasma membrane potential, it is important to note that the pHi is determined by multiple factors beyond those described above. Various transporters located at the plasma membrane and organellar membranes contribute to the regulation of pHi (Yenush 2016, Ariño et al. 2019). Among these, alkali/cation exchangers play a significant role in facilitating the exchange of protons and other cations, helping to regulate and balance the pHi and plasma membrane potential (Cyert and Philpott 2013, Ariño et al. 2019). Potassium (K^+^) is essential for various physiological processes in *S. cerevisiae*, including the maintenance of both pHi and plasma membrane potential, which are crucial for enzyme function, and the maintenance of cell volume, osmotic stability, negative charge compensation, protein synthesis, and overall cellular functioning (Cyert and Philpott 2013, Yenush 2016). Potassium plays key roles in organelles; the redistribution of this ion intracellularly is crucial for proper homeostasis maintenance. Most of the intracellular potassium is accumulated in the vacuole, whereas the concentration in the cytosol is rather low and kept constant (Herrera et al. 2013). The intracellular potassium distribution is dependent on pHi, which under optimal conditions is close to neutral in the cytosol, while in organelles such as the vacuole, endosomes, and Golgi apparatus, it is relatively more acidic compared to the cytosol, or slightly alkaline in the case of mitochondria (Orij et al. 2011, Ariño et al. 2019). Failure to maintain pH homeostasis has a substantial impact on the generation of the proton gradients required for proper potassium transport and distribution (Ariño et al. 2019). Disruptions in K^+^ homeostasis can impact the distribution and balance of other ions and lead to cellular dysfunction, highlighting the importance of finely tuned regulatory mechanisms. For instance, potassium has been shown to affect copper and iron metabolism (Zhang et al. 2019), as described in the next section. The potassium homeostasis regulation mechanisms involve complex networks of transporters and sensors that adjust K^+^ levels in response to environmental and internal cues, ensuring cellular adaptability under varying conditions, in particular under acetic acid stress. For instance, supplementation of K^+^ in the cultivation medium was found to enhance tolerance to acetic acid stress in *S. cerevisiae, Z. bailii*, and *Kluyveromyces marxianus* (Macpherson et al. 2005, Mira et al. 2010b, Xu et al. 2019b, Castillo-Plata et al. 2022, Antunes et al. 2023). Additionally, exposure to weak acids such as acetic, propionic, butyric, and benzoic acids was found to increase potassium influx and intracellular accumulation (Ryan et al. 1971, Macpherson et al. 2005).

To maintain a balanced membrane potential, *S. cerevisiae* relies on the coordination of potassium uptake and efflux systems; disruption of high-affinity potassium uptake results in plasma membrane hyperpolarization, whereas the absence of potassium efflux systems leads to plasma membrane depolarization (Kinclova-Zimmermannova et al. 2006, Navarrete et al. 2010, Petrezsélyová et al. 2011, Masaryk and Sychrová 2022). Despite yeast cells accumulating high concentrations of potassium (200–300 mM), excessive intracellular potassium levels can have a negative physiological impact besides destabilization of plasma membrane potential, such as vacuole deacidification, and alterations of cell volume and pHi (Cyert and Philpott 2013, Ariño et al. 2019). A comprehensive overview of potassium uptake and efflux systems in several yeast species is provided in (Ariño et al. 2019). In *S. cerevisiae*, the plasma membrane transporters Trk1 and Trk2 mediate high-affinity potassium uptake, which is the principal consumer of the electrochemical gradient generated by Pma1 (Portillo et al. 2005, Barreto et al. 2011). Trk1 is the main player in potassium uptake, as evidenced by the prominent increase in potassium requirements and growth impairment observed upon its deletion. Interestingly, the potassium affinity of Trk1 changes gradually from high to low according to intracellular and extracellular K^+^ concentration (Masaryk and Sychrová 2022). In contrast, the contribution of Trk2 to K^+^ acquisition is relatively minor, potentially attributed to its lower expression levels (Ramos et al. 1994, Yenush 2016), but this transporter exhibits a relevant role in the maintenance of plasma membrane potential (Petrezsélyová et al. 2011). Trk2 was initially suggested to be involved in low affinity transport, but subsequent findings demonstrated its ability to mediate high/moderate affinity potassium uptake when expressed from a strong promoter (Ramos et al. 1994, Yenush 2016). Notably, Trk1 has been associated to *S. cerevisiae*’s tolerance to acetic acid stress, as demonstrated by the inability of cells lacking this transporter system to grow under these conditions (Kawahata et al. 2006, Mira et al. 2010b, Mota et al. 2024). Additionally, mutations in *TRK1* have been shown to improve tolerance to acetic acid stress (Xu et al. 2019b). An interesting feature of Trk1 is its ability to mediate anion extrusion currents, in particular chloride (Cl^−^), but also other anions such as I^−^, Br^−^, SCN^−^, or NO_3_^−^, whose order of selectivity changes with pH (Kuroda et al. 2004, Rivetta et al. 2011). The currents and permeability of anions such as formate and acetate, tested at pH 5.5, are much smaller than the aforementioned anions (Kuroda et al. 2004, Rivetta et al. 2011). The implications, if any, this feature might have in the response and adaptation to acetic acid stress are elusive.

The regulation of Trk1 is complex (Ariño et al. 2019, Masaryk and Sychrová 2022) and has not been characterized under acetic acid stress. Under nonstressing conditions, it occurs mainly at the post-translational level through phosphorylation, with several key players contributing to its activity and stability. Notably, the kinases Hal4 and Hal5, from the Npr/Hal family (Antunes and Sá-Correia 2022), are known for stabilizing Trk1 at the plasma membrane by phosphorylating its C-terminal end, a process that also involves the activity of the kinase Snf1 (Portillo et al. 2005, Pérez-Valle et al. 2007, Casado et al. 2010). The Snf1 kinase is notable for its influence on the activity of the Trk system (Portillo et al. 2005). Deletion of *SNF1* results in several phenotypic effects, including reduced growth under potassium-limited conditions, decreased potassium accumulation, and hyperpolarization of the plasma membrane (Portillo et al. 2005). These effects can be partially mitigated by overexpression of *TRK1* and *HAL5* (Portillo et al. 2005), whereas overexpression of *SNF1* fails to suppress the growth defects of deletion mutants *trk1*∆*trk2*∆ and *hal4*∆*hal5*∆. These observations suggest that Snf1, in its nonphosphorylated state, as it occurs in the presence of glucose, acts upstream of the kinases Hal4 and Hal5 and the potassium importer Trk1 (Portillo et al. 2005). This implies that Snf1 plays a major role in modulating potassium uptake through a mechanism independent of the phosphorylation of T210. The phosphatase Ppz1 is implicated in the negative regulation of Trk1 by dephosphorylation. This action of Ppz1 is responsive to changes in pHi; an increase in pHi leads to Trk1 dephosphorylation, whereas a decrease in pHi triggers the interaction between Ppz1 and Hal3 (its main regulator), forming a complex that effectively inhibits Ppz1’s activity, resulting in the alleviation of the repression of Trk1 (Yenush et al. 2005, Albacar et al. 2022), consistent with an increase of its activity under weak acid stress (Xu et al. 2019b). *ARL1*, encoding a conserved guanine nucleotide-binding protein, has been identified as a determinant of acetic acid stress tolerance based on susceptibility phenotype observed in the deletion mutant strain (Kawahata et al. 2006, Mira et al. 2010b, Mota et al. 2024). Arl1 was initially proposed to act upstream of the kinases Hal4 and Hal5 in potassium uptake (Munson et al. 2004). However, subsequent studies have shown that deletion of *ARL1* does not affect Trk1’s intracellular localization (Munson et al. 2004, Pérez-Valle et al. 2007), suggesting an alternative mechanism of action. Arl1 is known to play a role in intracellular trafficking (Munson et al. 2004), which may be relevant in the context of acetic acid adaptation alongside the modulation of potassium fluxes. However, the precise roles of Arl1 in potassium uptake and acetic acid tolerance require further elucidation.

Potassium export is primarily ensured by three transport systems, Nha1, Ena ATPases, and Tok1 (Ariño et al. 2019). Tok1 is an outward-rectifier channel specific for potassium, which plays an important role in the maintenance of plasma membrane potential following depolarization (Maresova et al. 2006). Ena enzymes are P-type ATPases that couple the hydrolysis of ATP to the export of alkali metal cations. They are usually found in low levels under basal cultivation conditions and are weakly expressed at low pH (Ruiz and Arino 2007), thus they may not contribute substantially in the adaptation to weak acid-induced stress. Nha1 is a H^+^/K^+^,Na^+^ antiporter, being the plasma membrane potassium efflux system most biologically relevant for growth under low pH conditions. It is a housekeeping protein produced in low amounts and regulated at the post-translational level, whose main function is the detoxification of surplus alkali metal cations (Kinclová et al. 2001). It contributes to the regulation of plasma membrane and has a minor but significant role in the maintenance of pHi homeostasis (Sychrová et al. 1999, Kinclova-Zimmermannova et al. 2006). Nha1 and the endosomal K^+^,Na^+^/H^+^ antiporter Nhx1 are independently responsible and contribute equally for the efflux of potassium from the cytosol preventing overaccumulation of surplus potassium cations (Brett et al. 2005). Both the Nha1 and Nhx1 exchangers are determinants of tolerance to acetic acid stress and are required for cell growth at low pH values (Bañuelos et al. 1998, Brett et al. 2005, Mira et al. 2010b, Antunes et al. 2023). Moreover, Nha1 is a putative phosphorylation target of the Hrk1 kinase. Under acetic acid stress conditions, Nha1 has been proposed to have a relevant role in the maintenance of plasma membrane potential (Antunes et al. 2023). However, the mechanisms underlying its molecular function in acetic acid stress tolerance are still uncovered. Nhx1 has a dominant role relative to Nha1 in the modulation of pHi, by opposing the activity of the V-type H^+^-ATPase to alkalinize cellular compartments (Brett et al. 2005). The role of Nhx1 is not limited to ion homeostasis but also includes vesicle trafficking from the endosome, as alterations in pHi substantially impact organellar morphology and vesicular trafficking (Brett et al. 2005).

## Roles of heavy metal ions in the response and tolerance to acetic acid stress

### Acetic acid stress alters intracellular iron (Fe^2+^/Fe^3+^) availability

Metal metabolism undergoes significant alterations in yeast cells exposed to acetic acid stress. This stress condition disrupts the homeostasis of metal ions, particularly affecting iron levels and the expression of genes involved in iron uptake and metabolism, including the Aft1 transcription factor, which plays a major role in regulating the iron regulon under iron limitation conditions (Kawahata et al. 2006). Interestingly, a study has shown that under acetic acid stress, Aft1 exhibits increased transcription levels and is translocated to the nucleus, suggesting its activation (Kawahata et al. 2006). However, contrastingly, intracellular iron concentrations increase nearly 2-fold upon exposure to acetic acid stress (30 min), and abolition of the expression of genes involved in iron uptake exacerbates acetic acid sensitivity, even though supplementation with iron (1–100 µM of FeSO_4_) apparently does not alleviate acetic acid stress sensitivity. These seemingly contradictory findings may be consistent by considering that Aft1 activation does not depend on cytosolic iron levels but on the iron–sulfur (Fe–S) cluster (ISC) machinery and that ISCs are particularly vulnerable to oxidative stress, as their oxidation leads to loss of protein function and release of free iron (Lill et al. 2012). A decrease in ISC protein synthesis within mitochondria can activate the iron regulon even under iron-replete conditions (Lill et al. 2012), indicating that acetic acid stress might cause a reduction in ISCs’ availability. Thus, a possible regulation of iron uptake and metabolism, in response to a high sensitivity of ISCs to acetic acid-induced oxidative stress, is suggested since iron-containing proteins are involved in the oxidative stress response and iron excess may generate damaging oxygen radicals (Matsuo et al. 2017).

The protein kinase Snf1 activity is linked to ISCs sufficiency; a mechanism that ensures the inhibition of respiratory enzymatic activity in the absence of glucose when iron scarcity leads to insufficient availability of ISCs (Simpson-Lavy and Kupiec 2023). When there is competition among several cellular processes that require iron for proper function, the nuclear activity of Snf1 is inhibited, while its cytosolic activities remain unaffected. This inhibition is exerted by interaction of the protonated polyHIS tract of Snf1 with Aft1, which results in a decrease of nuclear Snf1 activity by half (Simpson-Lavy and Kupiec 2023). Under acetic acid stress conditions, it can be speculated that Snf1 exhibits a protonated polyHIS tract due to intracellular acidification, promoting its interaction with the nucleus-localized Aft1, thus resulting in inhibition of nuclear Snf1 activity (Simpson-Lavy and Kupiec 2023). The protein kinase Hog1, recognized as playing a role in acetic acid stress tolerance in *S. cerevisiae* and *Candida glycerinogenes* (Mollapour and Piper 2006, Mira et al. 2010b, Ji et al. 2016, Guaragnella et al. 2019, Ye et al. 2022, 2023), is rapidly activated through phosphorylation in response to this stress in *S. cerevisiae* (Mollapour and Piper 2006). Hog1 directly phosphorylates Aft1 at residues S210 and S224 (Martins et al. 2018), presumably leading to its reduced activity by promoting its nuclear export (Ueta et al. 2007). However, Aft1’s activity is not solely dependent on phosphorylation; its interaction with the monothiol glutaredoxins Grx3/4 is crucial for dissociating Aft1 from target promoters when iron levels are sufficient. This creates a scenario where cells with activated Hog1 might still retain active Aft1 (Martins et al. 2018). Therefore, it is plausible to speculate that acetic acid exposure could impair the activity of Grx3/4, which require Fe–S clusters as cofactor, and/or disrupt the interaction between Hog1 and Aft1, resulting in nuclear-localized active Aft1, and consequently, alterations in iron levels. Nevertheless, the mechanisms underlying the activation of Aft1, its interaction with Snf1 and Hog1, and the role of iron in yeast response and adaptation to acetic acid stress, require further studies.

Iron metabolism is intricately linked to potassium compartmentalization within organelles. Potassium uptake via the trans-Golgi network K^+^/H^+^ exchanger Kha1 is crucial for improving iron acquisition (Zhang et al. 2019). This process occurs through copper insertion into the multicopper ferroxidase apoFet3. Evidence supporting this includes observations that deletion of *KHA1* results in respiratory deficiency, a phenotype rescued by iron supplementation (Zhang et al. 2019). Moreover, the deletion of the potassium exchangers Vnx1 (vacuolar) or Nhx1 (late endosomal) results in cytosolic potassium accumulation, thereby facilitating copper loading to apoFet3 by increasing the K^+^ supply for Kha1. Conversely, deletion of the mitochondrial potassium transporter Mdm38 diminishes cytosolic potassium accumulation, resulting in reduced Golgi potassium levels and subsequent iron deficiency (Zhang et al. 2019).

In *C. albicans*, exposure to different weak acids, including acetic acid at pH 5.5, leads to elevated transcript levels of genes associated with iron homeostasis. However, contrasting with *S. cerevisiae*, a substantial reduction in intracellular iron levels is observed under acetic acid stress (4 h of exposure) (Cottier et al. 2015). This occurs despite the absence of iron limitation in the growth medium. However, enhancing iron uptake in *C. albicans* does not alleviate the reduction in intracellular iron levels or mitigate growth inhibition under weak acid stress conditions (Cottier et al. 2015). This suggests that the reduced intracellular iron level may not mainly occur due to impairment of iron uptake systems.

### Zinc supplementation alleviates yeast growth inhibition under acetic acid stress

Zinc (Zn^2+^) supplementation of the cultivation medium has emerged as a promising strategy to mitigate the inhibitory effects of acetic acid stress (Wan et al. 2015, Zhang et al. 2017, Chen et al. 2022). Zinc is an essential nutrient required for the structure and function of various proteins (Eide 2006). Addition of zinc sulfate to the culture medium of yeast cells exposed to acetic acid stress leads to increased levels of amino acids such as alanine, valine, and serine, with alanine accumulation being associated with enhanced glucose consumption and reduced accumulation of reactive oxygen species (ROS) and to the increase of intracellular glutathione (GSH), presumably as a response to oxidative stress (Wan et al. 2015). Zinc plays an essential role as a cofactor for proteins, including alcohol dehydrogenases (ADHs) the Cu, Zn superoxide dismutase (SOD) Sod1 and several transcription factors (Eide 2006). For instance, zinc is crucial for the proper functioning of the Haa1 transcription factor (Kim et al. 2019), as detailed in the next section. Under acetic acid stress, zinc supplementation leads to increased transcription levels of genes involved in ergosterol biosynthesis, accompanied by elevated ergosterol levels, and to decreased transcription levels of genes encoding acetate uptake transporters, such as Ady2, Ato2, and Jen1 (Zhang et al. 2017).

## Homeostasis and regulation of acetate fluxes in the response and tolerance to acetic acid stress

### Toxicity and adaptive responses to intracellularly accumulated acetate

Acetate is a by-product of yeast fermentation but can also serve as a carbon source. Under conditions of acetic acid stress, acetate accumulates within the cell, as explained above. The intracellular accumulation of acetate can disrupt cellular metabolism, leading to increased turgor pressure and significant oxidative stress (Pampulha and Loureiro-Dias 1990, Piper et al. 2001, Giannattasio et al. 2005). The presence of high acetate concentrations elevates the activity of antioxidant enzymes such as SOD and catalase, along with increasing levels of oxidatively modified proteins (Semchyshyn et al. 2011). Interestingly, as the result of preincubation with low concentrations of hydrogen peroxide, yeast cells can acquire cross-protection against acetic acid stress through the action of the transcription factor Yap1, underscoring the prooxidant effects of acetate (Semchyshyn et al. 2011). A *Candida krusei* strain with improved acetic acid tolerance obtained through genome shuffling was shown to display increased levels of intracellular catalase activity, also indicating that the accumulation of acetate results in oxidative stress in this species (Wei et al. 2008). The accumulation of acetate within the cell leads to an increase in misfolded proteins, adversely affecting their processing in the endoplasmic reticulum (ER). This can disrupt the synthesis of secretory and transmembrane proteins, including multidrug resistance (MDR) transporters (Kawazoe et al. 2017). In response to the elevated intracellular acetate levels and the resultant stress on protein folding in the ER, yeast cells activate the unfolded protein response (UPR) (Kawazoe et al. 2017). The UPR is a critical cellular mechanism to restore ER normal function by reducing the accumulation of misfolded proteins. It involves the Ire1 transmembrane RNase, which senses misfolded proteins in the ER (Kawazoe et al. 2017). Upon activation, Ire1 triggers the splicing of the transcription factor *HAC1* mRNA. Hac1 then enters the nucleus, where it upregulates the expression of genes involved in protein folding, ER-associated degradation (ERAD), and ER stress response (Kawazoe et al. 2017).

### Carboxylic acid uptake transporters mediate deleterious acetate uptake

The uptake of acetate in *S. cerevisiae* is facilitated through the carboxylic acid transporters Ady2 (Ato1), Ato2 (Fun34), and Jen1 (Casal et al. 2016, Zhang et al. 2017). Deletion of genes encoding these transporters, in particular *ADY2* and *ATO2*, results in improved growth under acetic acid stress (Gentsch et al. 2007, Zhang et al. 2017). Additionally, intracellular acetate accumulation in the *ady2*∆ deletion mutant was found to be reduced, suggesting that even in conditions where the extracellular pH is below acetic acid pKa, there may be some acetate uptake. The cytosolic accumulation of acetate is a signaling mechanism for the activation of the transcription factor Haa1 (Kim et al. 2019). This transcriptional regulator is the major activator of the transcription of acetic acid-responsive genes in *S. cerevisiae, C. glabrata*, and *Z. bailii* (Mira et al. 2010b, Bernardo et al. 2017, Antunes et al. 2018). The function of Haa1 in *S. cerevisiae* was shown to be dependent on the acetate binding to the N-terminal region, which converts Haa1 to an active form, likely through a conformational change, rendering it capable of DNA binding to target gene promoters (Kim et al. 2019).

### Acetate active export through MDR transporters

The adaptive response to acetic acid stress in *S. cerevisiae* involves the active export of acetate through specific plasma membrane transporters (Tenreiro et al. 2000, 2002, Dos Santos et al. 2014, Sá-Correia and Godinho 2022, Zhang et al. 2022). These transporters, implicated in multidrug/multixenobiotic resistance (MDR/MXR) belong to the major facilitator superfamily (MFS) or to the ATP-binding cassette (ABC) superfamily (Sá-Correia and Godinho 2022). The MFS transporters are secondary active transporters of small solutes, including sugars, amino acids, and drugs, across biological membranes using the energy stored in the electrochemical gradients (Pao et al. 1998). On the other hand, the ABC superfamily transporters utilize the energy of ATP hydrolysis to transport both small molecules and macromolecules across biological membranes, playing critical roles in cellular processes such as nutrient uptake, drug resistance, and detoxification (Pao et al. 1998). MDR/MXR transporters are documented as drug/xenobiotic efflux pumps for their hypothesized ability to catalyze the efflux of multiple cytotoxic compounds, thus contributing to the acquisition of stress tolerance in yeast cells. However, recent studies suggest that some MDR transporters may exert their effects indirectly, by modulating membrane potential and/or pHi, rather than through the direct export of cytotoxic compounds (Dos Santos et al. 2014). The physiological substrates for some of these transporters have been proposed, including the transport of the acetate metabolite (Fernandes et al. 2005, Zhang et al. (2022).

Among the various MFS transporters, some have been identified as determinants of tolerance to inhibitory compounds found in industrial processes, which translates to their ability to utilize the proton motive force to extrude toxic compounds from the cell in exchange for protons (Sá-Correia and Godinho 2022). Of these, Aqr1, Azr1, Tpo2, and Tpo3 have been shown to be determinants of acetic acid stress tolerance in *S. cerevisiae* (Tenreiro et al. 2000, 2002, Sá-Correia and Godinho 2022, Zhang et al. 2022). Deletion of the *TPO2* and *TPO3* genes in *S. cerevisiae* results in a significant increase in intracellular acetate accumulation, supporting the putative involvement of Tpo2 and Tpo3 in acetate efflux (Fernandes et al. 2005, Zhang et al. 2022). Both *TPO2* and *TPO3* genes are targets of the transcription factor Haa1 under acetic acid stress. The regulation of Tpo3 activity may involve Hrk1 since cells lacking this kinase exhibit increased acetate accumulation, and Tpo3 is a potential phosphorylation target of Hrk1 (Mira et al. 2010a, Guerreiro et al. 2017). However, this relationship remains to be elucidated. In *C. glabrata*, the transporters CgTpo3 and CgAqr1 are reported determinants of tolerance to acetic acid stress (Costa et al. 2013, Bernardo et al. 2017). Similarly to the *S. cerevisiae* homologues, CgTpo3, but not CgAqr1, is involved in the reduction of intracellular acetate (Tenreiro et al. 2002, Fernandes et al. 2005, Costa et al. 2013, Bernardo et al. 2017). CgAqr1 appears to be functionally like ScAqr1 and able to complement the acetic acid stress susceptibility phenotype of the *S. cerevisiae aqr1*∆ deletion mutant strain, suggesting an indirect role of CgAqr1 in acetic acid stress adaptation (Costa et al. 2013). Additionally, the transporter CgDtr1 from *C. glabrata* was identified as playing a role in pathogenesis and, as opposed to *S. cerevisiae* ScDtr1, contributes to acetic acid tolerance, functioning as a plasma membrane acetate exporter (Romão et al. 2017).

Regarding the ABC transporters involved in acetic acid tolerance, Pdr18 is the most well-characterized (Ribeiro et al. 2022). It plays a crucial role in actively incorporating ergosterol and maintaining maximum plasma membrane ergosterol content, especially to counteract the acetic acid stress-induced decrease of ergosterol content and lipid order (Godinho et al. 2018, Ribeiro et al. 2022). Pdr18 activity contributes to stabilizing plasma membrane potential and mitigate the increase in acetic acid-induced nonspecific membrane permeability (Godinho et al. 2018). Pdr12 is generally unresponsive to several stress factors, but it shows a strong affinity toward moderately lipophilic weak acids, providing tolerance to sorbic and benzoic acid, but not for short-chain fatty acids, acetic or formic acids (Nygård et al. 2014). Interestingly, deletion of *PDR12* in *S. cerevisiae* was shown to improve acetic acid stress tolerance, whereas its overexpression results in heightened sensitivity (Nygård et al. 2014). This phenomenon was considered the result of a higher conservation of ATP for other cellular processes when *PDR12* expression is abrogated (Nygård et al. 2014).

## Influence of plasma membrane lipid and protein alterations on ion fluxes under acetic acid stress

One of the key adaptation mechanisms to acetic acid stress in yeasts involves the modification of plasma membrane lipid composition. The rate at which the undissociated form of acetic acid diffuses into yeast cells is notably fast and is a process independent of the total concentration of acid (Lindahl et al. 2018). However, the presence of compounds in the culture medium that can partition into the plasma membrane, such as liposoluble compounds (e.g. octanoic and decanoic acids) and alcohols (e.g. ethanol and *n*-butanol) substantially impact the plasma membrane spatial organization and the diffusion rate of acetic acid and other weak acids (Viegas et al. 1989, Alexandre et al. 1996, Casal et al. 1998, Lindahl et al. 2018). In the case of ethanol, an inhibitory product of alcoholic fermentation, significant changes occur in plasma membrane physical properties, including an increase in the area per lipid molecule and a decrease in both membrane thickness and order, thus facilitating a faster diffusion of acetic acid across plasma membrane (Lindahl et al. 2018). Consequently, cells exposed to acetic acid in the presence of ethanol exhibit decreased specific growth rates and an extended lag phase of growth resultant from increased acetic acid diffusion rate, underscoring the combined toxic effects of ethanol and acetic acid on yeast cells (Lindahl et al. 2018).

The alteration of plasma membrane lipid composition constitutes an adaptive response to maintain plasma membrane physicochemical properties such as fluidity, thickness, permeability, and electrochemical potential under stress conditions (Lindberg et al. 2013, Lindahl et al. 2016, Godinho et al. (2018). The reduction in ergosterol levels (Lindberg et al. 2013, Godinho et al. 2018) and activation of sphingolipid synthesis (Lindberg et al. 2013, Guerreiro et al. 2016) has been observed in *S. cerevisiae* under acetic acid stress. The protective role of ergosterol against acetic acid stress presumably depends on its ability to limit the diffusion of acetic acid across the plasma membrane, a property that has been demonstrated in experiments using liposomes (Ferraz et al. 2023). However, it should be noted that the efficacy of ergosterol in reducing the permeability of plasma membrane to acetic acid is not only dependent on its concentration but also on the overall lipid composition of the plasma membrane (Gabba et al. 2020, Ferraz et al. 2023). The reported alterations in ergosterol concentration from yeast cells exposed to acetic acid varies, with studies reporting both decreases (Lindberg et al. 2013, Godinho et al. 2018) and increases (Guo et al. 2018) compared to unstressed cells. This variability highlights the complexity of sterol metabolism in response to environmental stresses, suggesting that culturing conditions, yeast growth phase, and level of acetic acid stress have a significant impact. Despite these alterations in ergosterol content under acetic acid stress, genes involved in ergosterol biosynthesis exhibit increased transcription levels and have been identified as determinants of tolerance to this stress (Mira et al. 2010a,b, Godinho et al. 2018, Guo et al. 2018, Zhang et al. 2017, Mota et al. 2024). Furthermore, increase in the transcription levels of the ABC transporter *PDR18* were shown to be coordinated with an increase of transcripts of genes involved in ergosterol biosynthesis, underscoring its relevance in the maintenance of physiological levels of ergosterol under acetic acid stress (Godinho et al. 2018).

The regulation of the sphingolipids’ biosynthetic pathway and sphingolipid content in *S. cerevisiae* and *Z. bailii* holds great influence on the tolerance to acetic acid stress (Guerreiro et al. 2016, Lindahl et al. 2016). In *S. cerevisiae*, proper regulation of sphingolipid metabolism is crucial for the activities of the plasma membrane and organellar H^+^-ATPases, as well as iron homeostasis (Tani and Toume 2015, Martins et al. 2018, Zhao et al. 2021). As previously mentioned, Pma1 localizes in sphingolipid-rich microdomains in the plasma membrane (Zhao et al. 2021). Additionally, deletion of genes involved in sphingolipid biosynthesis (*ELO3, ORM1*, and *ORM2*) leads to the reduction of V-ATPase activity (Tani and Toume 2015). Furthermore, deletion of *ISC1*, encoding a phospholipase involved in sphingolipid catabolism, results in the upregulation of the iron regulon, improper activation of Aft1, intracellular iron accumulation, and improved stress tolerance to high acetic acid concentrations (Rego et al. 2012, Martins et al. 2018).

Alterations in the levels of sphingolipid and its precursors impacts cellular processes such as cell fate, the activity of kinases and phosphatases, and protein trafficking (Rego et al. 2018). The activation of Ypk1, encoding a TORC2 activated protein kinase required for plasma membrane lipid and protein homeostasis, under acetic acid stress, results in the phosphorylation of several key proteins involved in sphingolipid biosynthesis (Guerreiro et al. 2016). Particularly, Ypk1 phosphorylates Orm1 and Orm2, which normally inhibit the enzyme complex l-serine:palmitoyl-CoA acyltransferase, the first committed step in sphingolipid biosynthesis. This phosphorylation relieves the inhibition on l-serine:palmitoyl-CoA acyltransferase, thus increasing flux into the sphingolipid pathway (Guerreiro et al. 2016). Additionally, Ypk1 phosphorylates Lac1 and Lag1, two functionally redundant isoforms of the ceramide synthase complex, diverting long-chain base (LCB) precursors more efficiently into ceramides for complex sphingolipid production under acetic acid stress (Guerreiro et al. 2016). Additionally, exposure to high acetic acid concentrations induces the translocation of Isc1 from the ER to the mitochondria, which promotes an increase in ceramide levels through the hydrolysis of complex lipids inducing mitochondria-mediated regulated cell death (Rego et al. 2018). Therefore, the tight balance of sphingolipid metabolism is crucial for the adaptation to acetic acid stress. In *Z. bailii*, a much higher basal concentration of complex sphingolipids than *S. cerevisiae* is found at the plasma membrane, which increases upon acetic acid stress exposure and translates to a higher impermeability to acetic acid (Lindberg et al. 2013, Lindahl et al. 2016). Underscoring the relevance of this adaptation mechanism for yeast tolerance to acetic acid stress, a *C. krusei* strain with improved tolerance to acetic acid was reported to exhibit higher plasma membrane impermeability (Wei et al. 2008).

## Conclusions and outlook

The response and adaptation to sublethal concentrations of acetic acid involves the complex coordination of mechanisms aimed at maintaining the physiological balance of asymmetric concentrations of various ions. This regulation is mediated by the coordinated activity of several membrane proteins, including the H^+^ pumps Pma1 and V-ATPase, alkali-cation exchangers, and acetate exporters, in addition to the remodeling of plasma membrane lipid and protein composition (Fig. 2).

Manipulation of ion concentrations, such as reported for K^+^ or Zn^2+^ supplementation in the growth medium (Macpherson et al. 2005, Mira et al. 2010b, Wan et al. 2015, Zhang et al. 2017, Xu et al. 2019b, Castillo-Plata et al. 2022, Chen et al. 2022, Antunes et al. 2023), emerges as a potential strategy that can be explored for enhancing yeast tolerance to acetic acid stress. Genetic engineering aimed at altering the composition of plasma membrane lipids and/or proteins has been explored as an approach to improve tolerance to acetic acid stress. For instance, overexpression of *PMA1* and mutations in *TRK1* have been demonstrated to confer improved tolerance to this stress (Lee et al. 2017, Xu et al. 2019b) and overexpression of genes involved in the synthesis and elongation of unsaturated fatty acids can increase the unsaturation index of fatty acids in the plasma membrane and elevate oleic acid levels in the cell, thereby providing protection against acetic acid stress (Zheng et al. 2013, Guo et al. 2018). Another study has attempted to increase the levels of complex sphingolipids in the plasma membrane through modulation of gene expression related to the production of LCB and very-long-chain fatty acids, as well as the conversion of ceramides into complex sphingolipids, but the approaches used have not yielded the desired outcomes (Lindahl et al. 2017). This underscores the complexity of the mechanisms underlying the regulation of sphingolipid metabolism and highlights the current challenges associated with engineering membrane lipid composition.

Understanding the mechanisms underlying tolerance to acetic acid stress, particularly concerning ion fluxes and balances, is crucial not only for developing more robust industrial yeast strains but also for identifying potential antifungal targets to mitigate the proliferation of drug-resistant pathogenic yeast strains. Future studies aimed at exploring the intricate mechanisms governing ion homeostasis and acetic acid tolerance in clinically relevant yeast species will be fundamental to develop effective antifungal therapies, given that numerous antifungal agents target ion homeostasis disruption and acetic acid has been implicated in the synergistic sensitization effects of azoles on *Candida* species (Moosa et al. 2004, Cunha et al. 2017, Li et al. 2018, Lourenco et al. 2019). Furthermore, delving into these mechanisms underlying ion homeostasis regulation and acetic acid stress tolerance in promising nonconventional yeast species of biotechnological relevance, such as *Yarrowia lipolytica* and *Rhodotorula toruloides* (Mota et al. 2022) could guide the development of superior yeast cell factories enhancing the sustainability, productivity, and efficiency of industrial bioprocesses, promoting a circular bio-based economy.
